# Reactive Navigation on Natural Environments by Continuous Classification of Ground Traversability

**DOI:** 10.3390/s20226423

**Published:** 2020-11-10

**Authors:** Jorge L. Martínez, Jesús Morales, Manuel Sánchez, Mariano Morán, Antonio J. Reina, J. Jesús Fernández-Lozano

**Affiliations:** Robotics and Mechatronic Lab, Andalucía Tech, Universidad de Málaga, 29071 Málaga, Spain; jesus.morales@uma.es (J.M.); manuel.sanchez.m@uma.es (M.S.); marianomoran@uma.es (M.M.); ajreina@uma.es (A.J.R.); jfl@uma.es (J.J.F.-L.)

**Keywords:** field navigation, ground vehicles, traversability classification, robotic simulation, 3D point cloud

## Abstract

Reactivity is a key component for autonomous vehicles navigating on natural terrains in order to safely avoid unknown obstacles. To this end, it is necessary to continuously assess traversability by processing on-board sensor data. This paper describes the case study of mobile robot Andabata that classifies traversable points from 3D laser scans acquired in motion of its vicinity to build 2D local traversability maps. Realistic robotic simulations with Gazebo were employed to appropriately adjust reactive behaviors. As a result, successful navigation tests with Andabata using the robot operating system (ROS) were performed on natural environments at low speeds.

## 1. Introduction

Reactivity is a necessary component for autonomous navigation in order to avoid obstacles present in the environment [1]. Unknown hazards on natural terrains can be found both above and below the ground level of the vehicle, which are commonly referred to as positive and negative obstacles, respectively [2,3,4].

Ground traversability should be continuously assessed by mobile robots to implement efficient motion planning [5] with limited computational resources [6,7]. If traversability results are very narrow, vehicle movements are unnecessarily restricted; on the other hand, if they are very permissive, the integrity of the robot is in danger [8].

Procedures for assessing terrain traversability can be specifically designed [9,10], but they can also be trained with real data [11,12] and by means of synthetic data [13,14]. This relevant analysis is usually performed with three-dimensional (3D) point clouds of the surroundings acquired from an on-board sensor [8,15].

Depth data for traversability can be acquired with stereo [12] or time-of-flight cameras [16]. Farther ranges can be obtained by combining successive two-dimensional (2D) laser scans while the vehicle advances [17,18,19], or by a 3D laser rangefinder. In the latter case, the sensor can be a costly commercial multibeam model [4,20] with high scan frequency, or a more affordable actuated 2D scanner, which admits higher resolution but requires more acquisition time [21,22,23].

Point clouds as input data for ground-vehicle navigation can be directly used and immediately discarded [24,25] or they can be incrementally stored using simultaneous localisation and mapping (SLAM) to be employed later [26]. Generally, the last option implies building and maintaining an explicit representation of the environment via a 3D global map [23].

This paper pursues to enhance our previous work [25] about unmanned navigation at low speeds with mobile robot Andabata, which carries an actuated 2D laser scanner as its main exteroceptive sensor. To this end, a previously trained classifier was used to analyse point traversability of levelled 3D depth data acquired in motion [14]. This reliable ground assessment was employed to continuously build local 2D traversability maps for reactive operation. Realistic robotic simulations were used to appropriately tune reactive parameters before testing waypoint navigation with localisation uncertainty on the real robot.

The rest of the paper is organised as follows. Section 2 highlights the main contributions of the paper in relation to its most related works. Then, Section 3 describes the simulation of Andabata on Gazebo [27] that was used to tune reactive navigation, of which the scheme is proposed in Section 4. Simulated and real experiments are discussed in Section 5 and Section 6, respectively. Lastly, conclusions, acknowledgements, and references complete the paper.

## 2. Related Works

Reactive behaviors are commonly used by ground vehicles to avoid local obstacles on rough [28] and vegetated [29] terrain, or during disaster scenarios [26] or planetary exploration [6,30,31], while trying to achieve previously planned goal points. In this context, the risk or interest associated with the immediate movements of the vehicle, such as straight lines [17,25], circular arcs [3,30,32], or both [6], should be evaluated to produce adequate steering and speed commands [24,33].

Motion planning and traversability assessment can directly occur on the 3D point cloud [15,20] or on a compact representation of 3D depth data, such as a 2.5D elevation map [21,28,30] or a 2D horizontal grid [32,34]. Roughness and terrain slopes are usually considered to evaluate the traversability of 2.5D maps [17,31]. The cells of 2D maps may contain fuzzy traversability data [33] or precise occupancy values such as free, obstacle, or unknown [34].

Robotic simulation platforms that include a physics engine such as V-REP [35] or Gazebo [27] allow for obtaining realistic information of a ground vehicle moving on its environment. Thus, they can be employed to evaluate elementary motions [28], assess traversability [13,20], or hand-tune navigation parameters [26].

Most field-navigation components that we developed for mobile robot Andabata [25] with the robot operating system (ROS) [36] were kept in this paper. The main difference with our prior work is that we now use a terrain-traversability classifier instead of fuzzy elevation maps, of which the main drawback is requiring greater processing times than the acquisition times of individual 3D scans, which causes some of the acquired 3D scans to not be processed for navigation.

In this paper, linear movements for reactive navigation are evaluated over a 2D polar traversability grid built by projecting onto it classified points from a levelled 3D scan acquired with local SLAM. Robotic simulations with Gazebo [27] were employed to test reactivity before real tests. Traversability is individually assessed for each Cartesian point with a random-forest classifier from the machine-learning library *Scikit-learn* [37]. This estimator was previously trained with synthetic data providing the most accurate results for real data from Andabata among other available classifiers from this freely available library [14].

Although the paper maintains many points in common with related works, two original contributions are highlighted:

- The use of 2D polar traversability maps based on 3D laser scans classified point by point for selecting motion directions on natural environments.
- The employment of extensive robotic simulations to appropriately tune reactivity before performing real tests with uncertain global localisation.

## 3. Mobile Robot Simulation

**Figure 1:**
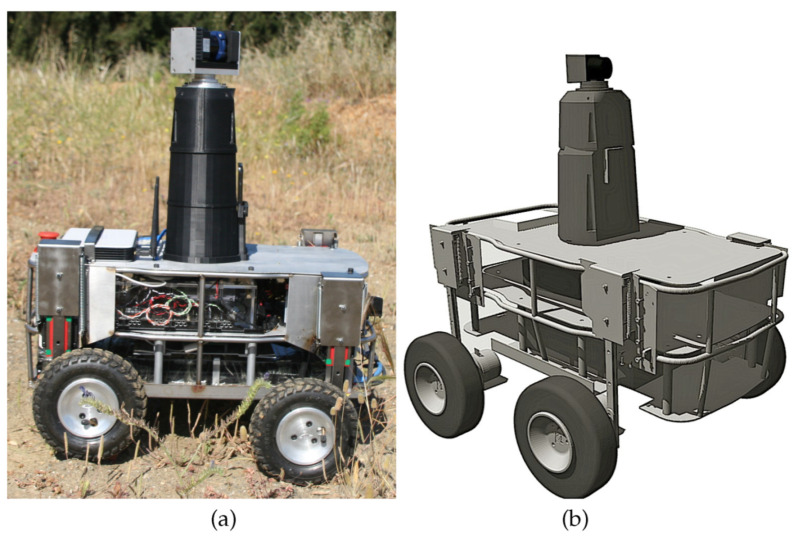
Andabata mobile robot: (**a**) Photograph on irregular terrain; (**b**) model in Gazebo.

Andabata is a skid-steered vehicle that weighs 41 kg, is 0.67 m long, 0.54 m wide, and 0.81 m tall (see Figure 1a). The components of Andabata were modelled in Gazebo [27] with different links and joints (see Figure 1b).

The main chassis of the robot, which contains the battery, the motor drivers (two 2 × 32 Sabertooth power stages connected to two 2 × Kangaroo controllers), and the computer (16 GB RAM, Intel Core processor i7 4771 with 4 cores at 3.5 GHz, and 8 MB cache) [25], was modelled in detail with Gazebo (see Figure 1b).

The complete navigation system of Andabata was fully implemented on the on-board computer under the ROS [36]. This software can be simulated in Gazebo through a set of ROS packages called *gazebo_ros_pkgs* (http://wiki.ros.org/gazebo_ros_pkgs) that provide the necessary interfaces by using ROS messages and services, and to build different Gazebo *plugins* for sensor output and motor input. In this way, it is possible to interchangeably test the same ROS nodes on the real robot and on the simulator.

Each of the four wheels of the 10 cm radius is connected to its own gear train, DC motor, and encoder through a revolute joint. All these locomotion elements, in turn, are linked with the main chassis through a prismatic joint to emulate the passive suspension of the vehicle with two springs and a linear guide with a stroke of 6.5 cm [25]. The suspension model in Gazebo assumed rigid wheels, an elasticity constant of 3976.6 N m^−1^, and a damping coefficient of 75.76 kg s^−1^.

An approximate kinematical model that exploits the equivalence between skid steering and differential drive was used for this robot [38]. The symmetrical kinematic model relates the longitudinal and angular velocities of the vehicle (*v* and ω, respectively) with the left and right tread speeds measured by the encoders ($v_{l}$ and $v_{r}$, respectively) as:

(1) $$\begin{array}{ccc} v & = & \frac{v_{l}+v_{r}}{2}, \end{array}$$

(2) $$\begin{array}{ccc} \omega & = & \frac{v_{r}-v_{l}}{2y_{ICR}}, \end{array}$$

where $y_{ICR}=0.45\;m$ is the mean value of the instantaneous centers of rotation (ICR) of the treads [25]. On the other hand, control inputs $v_{l}^{sp}$ and $v_{r}^{sp}$ could be obtained from setpoint velocities for vehicle $v^{sp}$ and $\omega^{sp}$ as:

(3) $$\begin{array}{ccc} v_{l}^{sp} & = & v^{sp}-y_{ICR}\;\omega^{sp}, \end{array}$$

(4) $$\begin{array}{ccc} v_{r}^{sp} & = & v^{sp}+y_{ICR}\;\omega^{sp}. \end{array}$$

If any of the control inputs exceeded its limits of $v_{max}=\pm 0.68\;m\;s^{-1}$, setpoint velocities were divided by positive factor

(5) $$e=\frac{\left|v^{sp}\right|+y_{ICR}\;\left|\omega^{sp}\right|}{\left|v_{max}\right|}$$

to maintain desired turning radius $r^{sp}$ for the vehicle:

(6) $$r^{sp}=\frac{v^{sp}}{\omega^{sp}}=\frac{v^{sp}/e}{\omega^{sp}/e}.$$

Thus, the maximal linear velocity of vehicle $v_{max}$ can only be achieved during a straight-line motion, and approaches zero as $r^{sp}$ reduces [25]. The response of tread speeds ($v_{l}$ and $v_{r}$) to speed commands ($v_{l}^{sp}$ and $v_{r}^{sp}$) from the computer is not instantaneous, and it was modelled in Gazebo as a first-order system with a time constant of 35 ms.

A column was attached on top of the main chassis and centered (see Figure 1b). On the front side of the column, a rectangular cuboid was fixed to represent the on-board smartphone of Andabata that contained a global-positioning-system (GPS) receiver (with a horizontal resolution of 1 m), inclinometers, gyroscopes, and a compass [25]. Data from gyroscopes and inclinometers can be directly obtained from the default physics engine of Gazebo (open dynamics engine, ODE). GPS and compass data can be obtained by adding Gaussian noise to the exact position and heading of the mobile robot on the virtual environment, respectively. *Hector_gazebo_plugins* (http://wiki.ros.org/hector_gazebo_plugins) were employed to incorporate all these sensors to Gazebo with their corresponding acquisition rates.

The Gazebo model of two-dimensional (2D) laser scanner Hokuyo UTM-30LX-EW was connected to the top of the column (see Figure 1b) through a revolute joint to emulate the 3D laser rangefinder of Andabata [39], which was based on the unrestrained rotation of this 2D sensor around its optical center [40]. The 2D scanner has a field of view of 270°, a resolution of 0.25°, ±3 cm accuracy, and a range of measurements from 0.1 to 15 m under direct sunlight.

**Figure 2:**
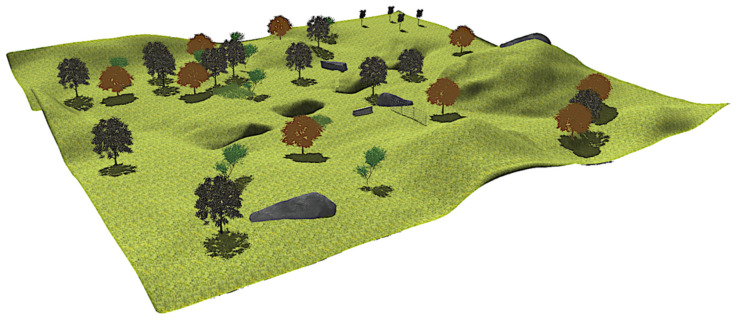
General view of natural environment built with Gazebo.

Figure 2 displays a general view of the natural environment generated with Gazebo [39], which was a square of a 50 m side where Andabata navigated. It contained many positive obstacles, such as high grass, big rocks, trees, a fence, and a barrier. It also had several ditches that acted as negative obstacles.

**Figure 3:**
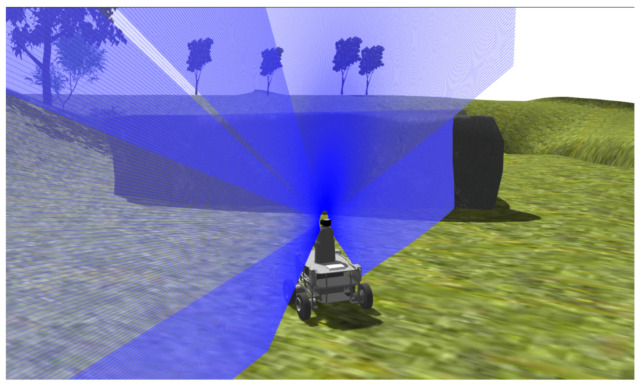
Gazebo simulation of Andabata moving on environment. Blue lines represent the acquisition of a single 2D vertical scan.

Figure 3 shows the simulation of Andabata moving over the environment. The acquisition of one of the 2D vertical scans that compose a full 3D scan is represented with blue lines. Thick blue lines indicate detected ranges, whereas thin lines represent no measurement. The horizontal resolution of the 3D rangefinder depends on the turns made by the entire 2D sensor and by its turning speed. The blind region of the 3D sensor is a cone that begins at its optical center (h = 0.73 m above the ground) and includes the complete robot below.

## 4. Reactive-Navigation Scheme

The global navigation objective consists of visiting an ordered list of distant waypoints moving at a constant linear velocity $v^{sp}$ [25]. The proximity radius around the current waypoint used to commute to the next was reduced from 10 to 3 m due to improved GPS accuracy of the on-board smartphone.

For local navigation, the 3D laser rangefinder has been configured to provide a full 3D scan of the surroundings every $t_{s}=3.3\;s$ with 32,000 points approximately, while Andabata moves. The whole point cloud is levelled by using local 3D SLAM without loop closures [25] and it is referred to the place where it began its acquisition.

Then, traversability is assessed for individual points with a random-forest classifier [14]. For every scan, a 3D tree data structure is built, and three spatial features for every point are deduced from its five closest neighbors. Indefinite points are those with fewer than five neighbors. In this way, every point below 12 m from the center of the 3D scan is individually classified as traversable, nontraversable, or indefinite. This processing takes approximately $t_{c}=1.23\;s$ per each 3D scan, almost all this time being for feature extraction.

**Figure 4:**
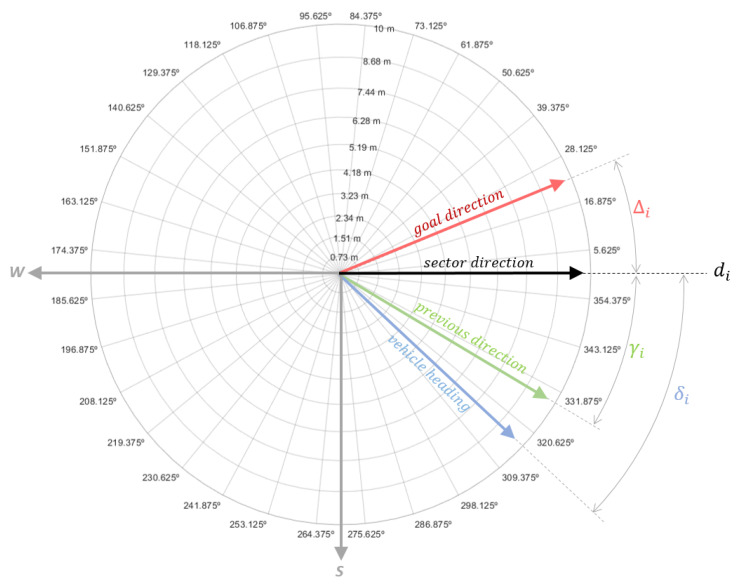
Goal-direction match for sector direction $d_{i}$.

Once the 3D scan is classified, a 2D traversability map is built by projecting every 3D point on a horizontal plane centered at the current position of the robot (which is different from the center of the 3D scan because of robot motion during 3D scan acquisition). The navigation map consists of a polar grid divided into 32 sectors of 11.25° and nine annuluses formed by ten successive uneven radius *r*:

(7) $$r^{j}=10\frac{\tau^{j}-1}{\tau^{10}-1},\;j=1\cdots 10,$$

where expansion ratio τ=1.0682 allows for a growing radius from *h* to 10 m (see Figure 4). All local maps are aligned with the west and south at 180° and 270°, respectively.

Then, every cell inside the 2D grid, with the exception of the central circle of radius *h*, is labelled depending on the projected points that fell inside as follows:

- If the cell does not contain any point at all, it is labelled as empty in white.
- With at least 15% of nontraversable points, the cell is classified as nontraversable in red.
- With more than 85% of traversable points, the cell is labelled as traversable in green.
- In any other case, the cell is classified as indefinite in grey.

All lines $d_{i}$ from the center of every sector *i* are checked as possible motion directions for Andabata. Selected direction $d_{j}$ is the one that minimises the cost function:

(8) $$J\left(d_{i},i\right)=\frac{G(d_{i})}{T(i)},\;i=1\ldots 32,$$

which considers both goal-direction match *G* and sector traversability *T*.

Goal-direction match *G* for every $d_{i}$ is calculated as

(9) $$G\left(d_{i}\right)=|\Delta_{i}|+k_{1}\;|\delta_{i}|+k_{2}\;\left|\gamma_{i}\right|,$$

where $k_{1},\;k_{2}$ are adjustable gains, and $\Delta_{i}$, $\delta_{i}$, and $\gamma_{i}$ are the angular differences between $d_{i}$ with respect to the goal direction, the current heading of the vehicle, and the previous motion direction, respectively (see Figure 4).

**Figure 5:**
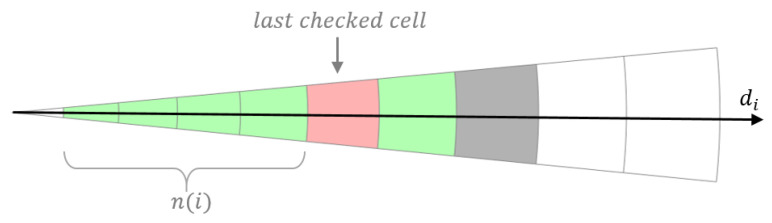
Traversability evaluation for sector *i*.

Traversability *T* for every sector *i* is computed as

(10) $$T\left(i\right)=k_{3}\;\left(1+n\left(i\right)\right)+k_{4}\;\left(n\left(i+1\right)+n\left(i-1\right)-|n\left(i+1\right)-n\left(i-1\right)|\right),$$

where n(i) is the number of traversable cells on sector *i* from inside out until a nontraversable cell or the outer cell is reached (see Figure 5), and $k_{3}$, $k_{4}$ are adjustable gains that reward clear directions on the sector and on its two adjacent sectors, respectively.

To sum up, it is necessary to adjust navigation parameters $k_{1},\;k_{2},\;k_{3}$, and $k_{4}$ of cost function *J*. Direction evaluation is relatively simple and only takes approximately $t_{m}=0.15\;s$ on the on-board computer.

Lastly, steering commands $\omega^{sp}$ for Andabata are computed every time that the vehicle heading is updated at a rate of 50 Hz by the compass:

(11) $$\omega^{sp}=g\;\delta_{j},$$

where g=1 is a proportional gain that controls the heading change of the vehicle to achieve selected direction $d_{j}$.

**Figure 6:**
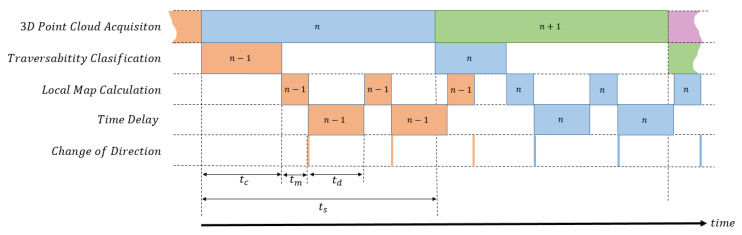
Task schedule for reactive navigation of Andabata.

Figure 6 shows the task schedule for local reactive navigation. Time delay $t_{d}$ is intentionally introduced to provide set points for steering three times per each 3D laser scan by building three 2D traversability maps approximately every $t_{s}/3=1.1\;s$. For this purpose, the delay should fulfil

(12) $$t_{m}+t_{d}\approx t_{s}/3\Rightarrow t_{d}\approx 0.95\;s.$$

Nevertheless, the interval between changes of direction is not constant because $t_{c}$ heavily depends on the number of points of each 3D scan. Figure 6 also shows that the acquisition of a levelled 3D scan simultaneously occurs with the classification of a previous point cloud and the traversability map calculation by executing in parallel ROS nodes on different cores of the computer processor.

## 5. Simulated Experiments

The reactive strategy for local navigation was extensively tested with Gazebo simulations to adjust its four parameters. The main one is $k_{3}$, which regulates how the pursue-goal and the obstacle-avoidance behaviors combine. Parameters $k_{1}$ and $k_{2}$ try to avoid changes of direction, and $k_{4}$ tries to favor free courses. As a result of a trial-and-error process, the following parameters were manually selected: $k_{1}=k_{2}=0.15$, $k_{3}=1$, and $k_{4}=0.3$.

**Figure 7:**
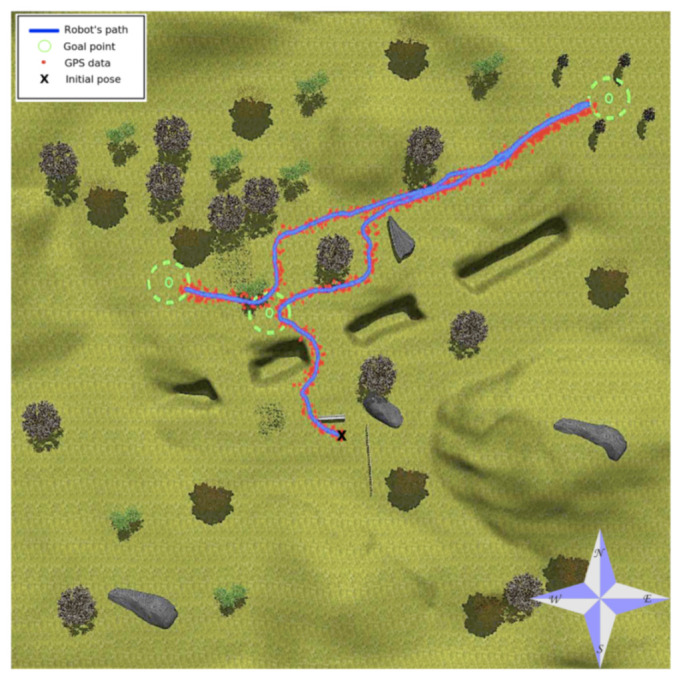
Aerial view of path followed by Andabata on the environment.

Figure 7 shows with a blue line the global path followed by Andabata while pursuing three distant waypoints on the generated environment with $v^{sp}=0.3\;m\;s^{-1}$. In this figure, GPS data are plotted with red dots, goal points are drawn with a small green circle surrounded by a proximity green circle of 3 m, and a black X marks the beginning of the path.

Figure 7 shows that the reactive component of the navigation system allows for avoiding both positive and negative obstacles. Concretely, in the way to the first goal, Andabata avoided a barrier and a deep ditch. Then, when trying to reach the second goal, it circumnavigated a tree and a big rock. Lastly, it eluded tall grass in the vicinity of the last goal.

**Figure 8:**
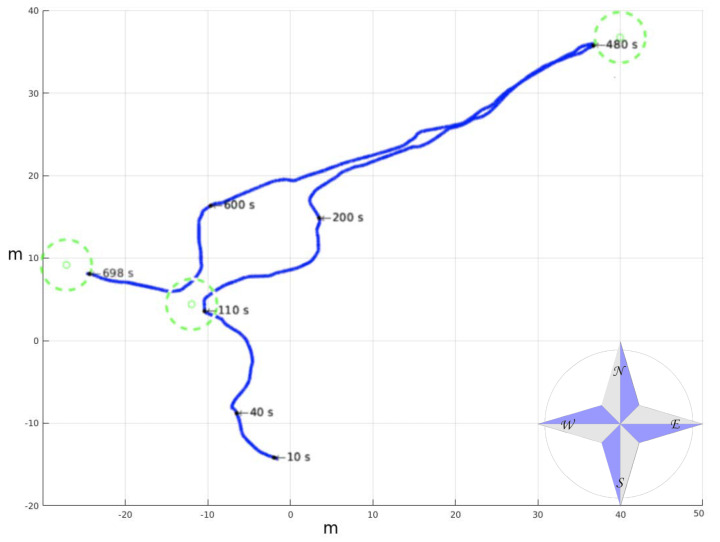
Trajectory followed by Andabata with time stamps.

**Figure 9:**
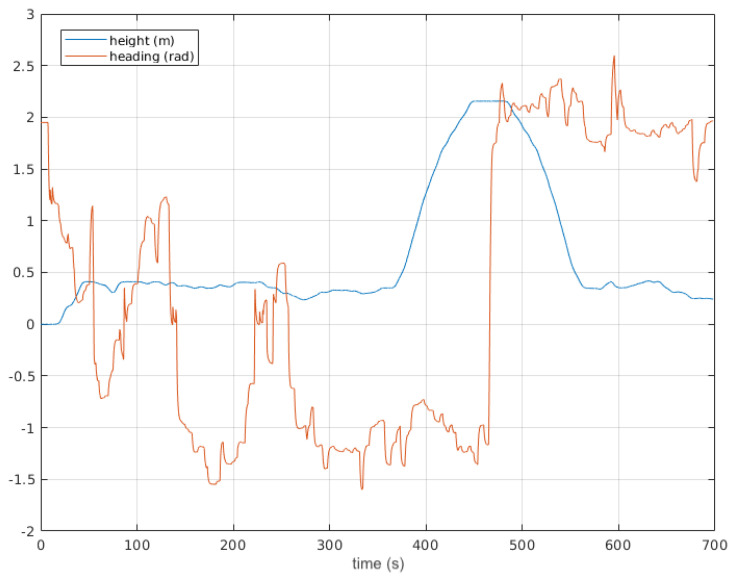
Vehicle elevation and heading during autonomous navigation.

Figure 8 contains the 161 m length trajectory of Figure 7 with time stamps and horizontal coordinates. In total, 186 3D point clouds were acquired, and their corresponding 558 2D local traversability maps were built. The elevation and heading of the vehicle along this trajectory are represented in Figure 9. Smooth heading changes can be observed with the exception of the 180° turn when the second goal was reached at 480 s. Moreover, the maximal height climbed and descended by Andabata was 2.15 m in total.

**Figure 10:**
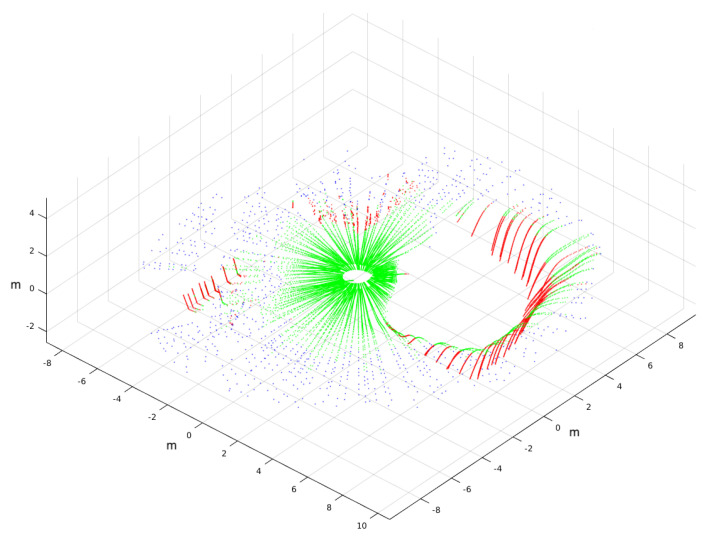
Simulated 3D point cloud near ditch classified by traversability.

An example of a 3D scan classified by traversability is shown in Figure 10. This levelled point cloud was acquired on the way to the first goal near the ditch. Traversable, nontraversable, and indefinite points are represented in green, red, and blue, respectively.

**Figure 11:**
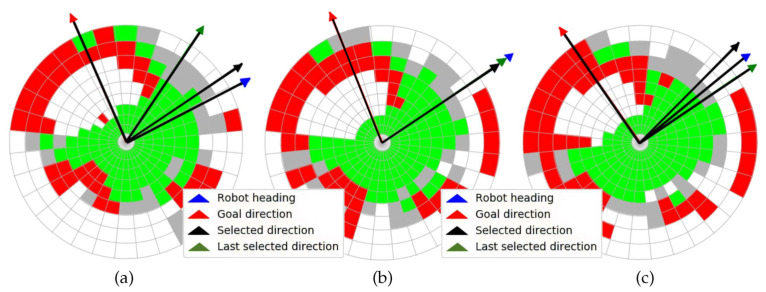
(**a**) First, (**b**) second, and (**c**) third traversability maps built for 3D point cloud of Figure 10.

The three consecutive traversability maps built with the 3D scan of Figure 10 are represented in Figure 11. The ditch appears on these maps as a large white region on the left and up that is crossed by the goal direction on the northwest. Nevertheless, the selected direction kept the robot far from this negative obstacle, as can be observed in the three maps, where the robot heading was pointing northeast.

A demonstration of this robotic simulator was publicly presented during the European Robotics Forum 2020 (https://www.eu-robotics.net/robotics_forum/). Reactive navigation was tested performing a live cyclic experiment with the same initial and final waypoints. Nevertheless, the 2D traversability maps to decide motion directions were always changing because 3D laser scans never coincided for the same places.

## 6. Andabata Experiments

Once the parameters of the reactive controller had been adjusted via simulations, they were tested with Andabata on a trail in a hollow and carless urban park.

### 6.1. Trail in a Hollow

**Figure 12:**
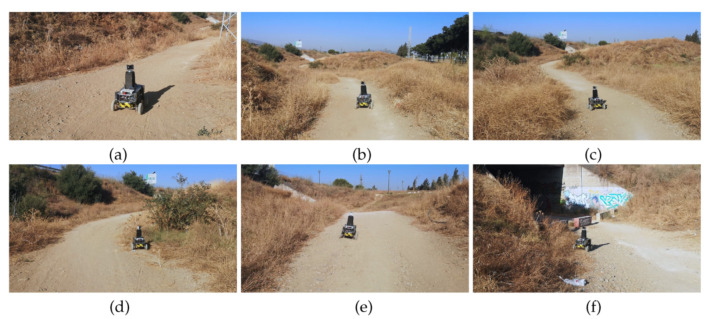
Andabata moving on the trail inside the hollow. Successive photographs shown from (**a**) beginning to (**f**) end of the trajectory.

Two waypoints have been chosen to follow a trail with inclines inside a hollow. In general, the borders of the trail consisted of dry weeds and hills (see Figure 12).

**Figure 13:**
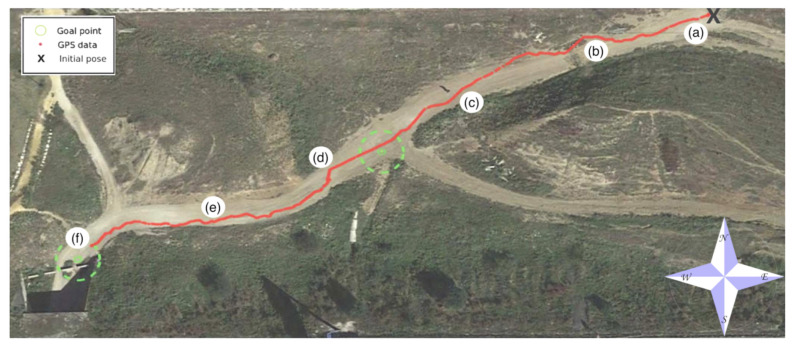
GPS measurements during autonomous navigation on the hollow. Locations where each photograph of Figure 12 was taken are indicated.

Figure 13 shows an aerial view of the path followed by Andabata as recorded by GPS data. With $v^{sp}=0.3\;m\;s^{-1}$, the trajectory was 133 m long and lasted 462 s. Altogether, 137 3D scans were acquired with an average value of 27,694 points.

**Figure 14:**
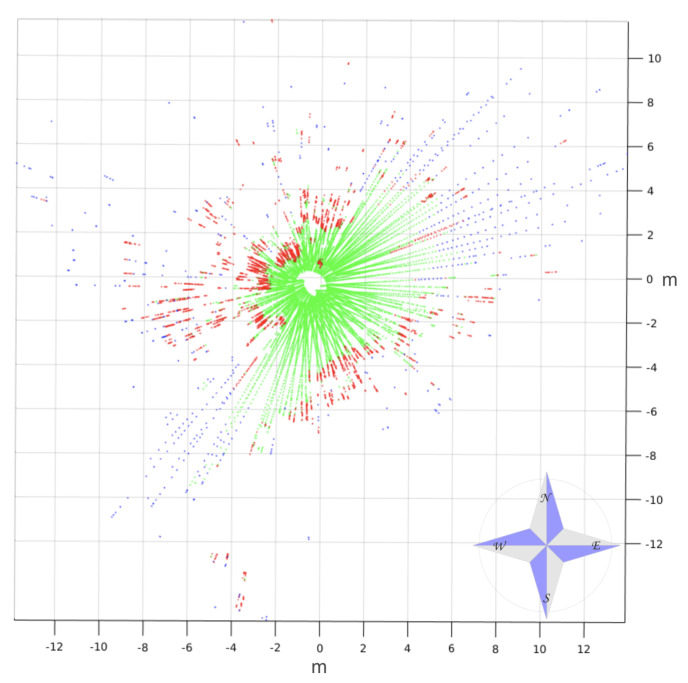
Top view of real 3D scan on trail classified by traversability.

**Figure 15:**
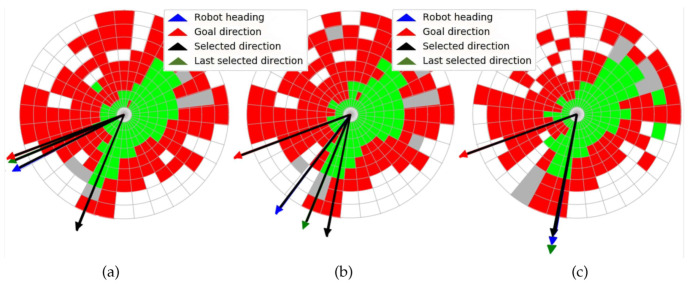
(**a**) First, (**b**) second, and (**c**) third traversability maps generated for real 3D scan of Figure 14.

A top view of a real 3D point cloud classified by traversability is shown in Figure 14. This particular scan was acquired on the way to the second goal, a few meters after leaving the first goal (see Figure 12d). The three consecutive traversability maps built from this 3D scan are represented in Figure 15. A hill and sparse vegetation appeared in the direction to the second goal, so the robot had to deviate from its current direction, as could be verified by the heading change between the first and successive traversability maps.

### 6.2. Park Course

**Figure 16:**
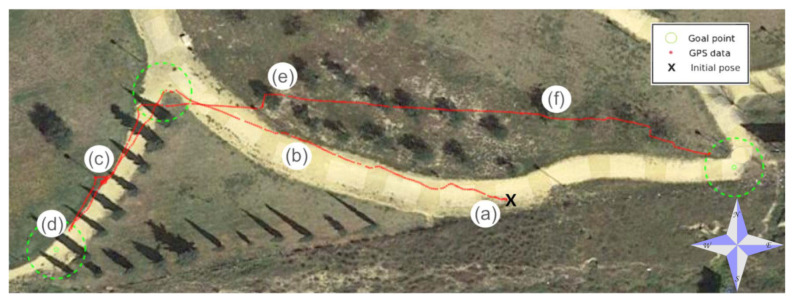
GPS measurements during unmanned navigation in the park. Locations that correspond to each photograph of Figure 17 are indicated.

Unmanned navigation on a careless urban park was also tested using three goal points (see Figure 16). The intermediate point appeared twice on the way to the extreme points on the west and the east. The aerial view of Figure 16 shows the GPS trajectory when Andabata was commanded with $v^{sp}=0.3\;m\;s^{-1}$.

**Figure 17:**
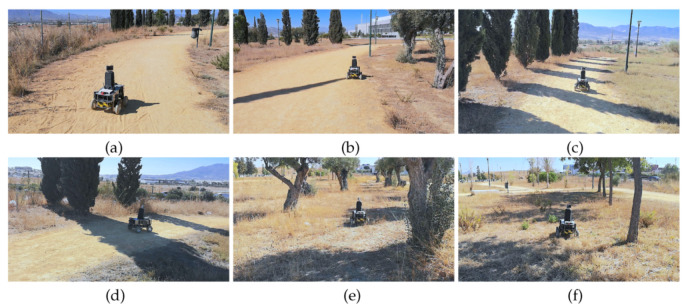
Photographs of Andabata on the park from (**a**) beginning to (**f**) end of the trajectory.

The almost plain surface of the park contained both natural (trees, bushes, and weeds) and artificial obstacles (lamp-posts, fences, and rubbish bins). Most of the trajectory was followed over the yellow course with the exception of the last stretch, where the robot went through a rough zone with trees and weeds pursuing the last goal point to the east (see Figure 17).

The park trajectory was 181 m long and lasted 660 s. In total, 183 3D scans were acquired by Andabata. The average value of 34,498 points per 3D scan was greater than that in the previous experiment because sky visibility was reduced, mainly due to treetops.

### 6.3. Discussion

**Figure 18:**
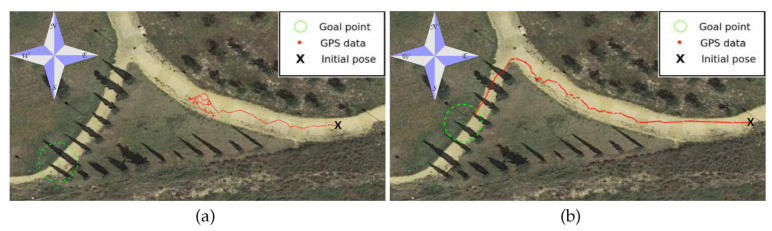
Reactive navigation in urban park without intermediate waypoint: (**a**) failure and (**b**) success.

Waypoint selection is very important to complete navigation goals. To test it, we repeated the park course by eliminating the intermediate point. Nevertheless, Andabata failed to reach the western goal (see Figure 18a). This failure was because the robot did not find a path to the goal though the weeds, and kept turning around.

In this case, there was a conflict of behaviors in the reactive controller: if the vehicle advances on a free-of-obstacles direction $d_{i}$ over the yellow course, it increases the angular difference with respect to goal direction $\Delta_{i}$. Thus, high $G(d_{i})$ (9) and T(i) (10) values were obtained at once in the numerator and denominator of cost function $J(d_{i},i)$ (8), respectively. In fact, if the western goal point were moved a few meters to the northeast, the robot succeeded in reaching it by circumnavigating weeds and pine trees (see Figure 18b).

Generally, an unmanned vehicle should overcome water bodies during a cross-country course to prevent electrical damage or becoming stuck inside [41]. Deep-water elements can be indirectly detected with a 3D laser scanner by lack of measurements related with laser-beam deflections that make them behave like negative obstacles [4].

**Figure 19:**
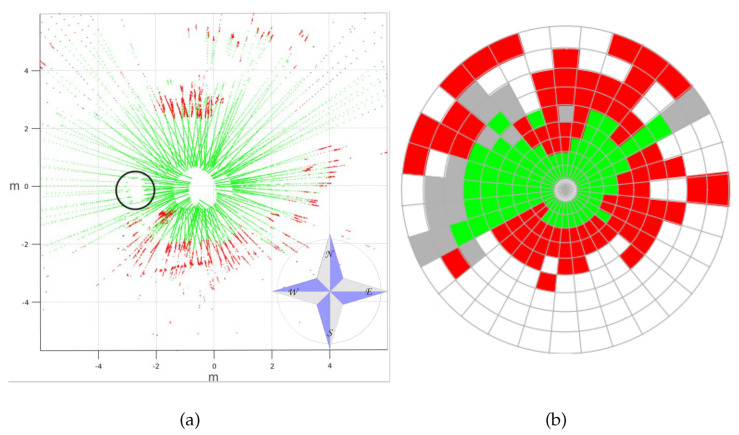
(**a**) Close view of 3D point cloud from top that contains a puddle in front of the vehicle indicated by black circle and (**b**) its first traversability map.

However, Andabata failed to avoid puddles that it encountered on its way because it is sufficient to have a point classified as traversable inside a cell near a puddle on the traversability map to label the almost-empty cell as green (see Figure 19).

**Figure 20:**
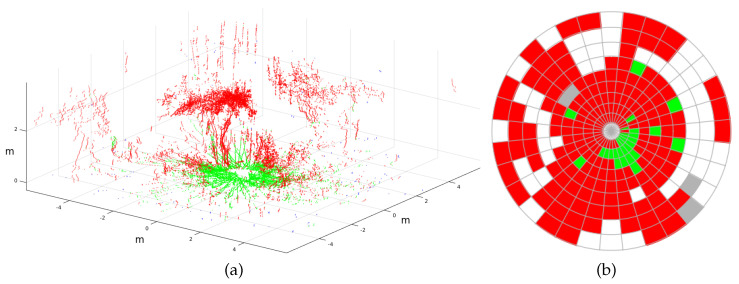
(**a**) Classified 3D laser scan with Andabata under a tree and (**b**) its first traversability map.

Another relevant issue for outdoor navigation is overhangs such as tree canopy or tunnels [42]. Figure 20 shows a 3D point cloud taken from the urban-park experiment where the robot was close to an olive tree. Tall points from the treetop were correctly classified as nontraversable in red. However, the projection of these points on the 2D traversability map caused most ground cells around the vehicle to be considered nontraversable, considerably reducing the free space.

Dynamic obstacles such as animals can also be found by a vehicle on natural environments. With this in mind, we tested navigation while Andabata crossed with a pedestrian. However, the robot was not able to properly prevent collision because the acquisition rate of 3D scans (i.e., $t_{s}=3.3\;s$) was clearly insufficient for this purpose.

## 7. Conclusions

This paper described the case study of mobile robot Andabata that distinguished traversable ground from 3D point clouds acquired in motion of its vicinity by using a supervised–trained classifier. A reactive navigation scheme at low speeds was proposed to achieve waypoints with uncertain GPS localisation while avoiding static obstacles on natural terrains.

Realistic robotic simulations with Gazebo were employed to appropriately adjust reactive parameters. In this way, numerous experiments with Andabata were avoided, which was a considerable gain in testing time and robot integrity. Field experiments were presented where different paths were successfully followed by Andabata with the ROS by using only a few distant waypoints.

This paper enhanced our previous work [25] about autonomous navigation with Andabata. This was achieved by processing all 3D laser scans acquired by the robot in motion. Moreover, reactivity is improved by building three 2D traversability maps for every levelled 3D point cloud as the robot moved through it.

There was less free space in real scenarios than in the simulated environment, which means that it would be convenient to work with a more complicated simulated scenario by including more elements, such as weeds and puddles. This is a matter for future improvements, and to better label the cells of the 2D traversability maps to consider small negative obstacles and discard tall overhangs.

Future work also includes discerning when the robot gets stuck, detecting dynamic obstacles in front of the vehicle by processing images from the camera of the on-board smartphone, and the automatic learning of the proposed reactive parameters by reinforcement learning [43] through Gazebo simulations.

## Abbreviations

The following acronyms are used in the manuscript:

2D	Two-dimensional
2.5D	Two-and-a-half-dimensional
3D	Three-dimensional
GPS	Global positioning system
ICR	Instantaneous center of rotation
ODE	Open dynamics engine
RAM	Random access memory
ROS	Robot operating system
SLAM	Simultaneous localisation and mapping
USB	Universal serial bus
